# mHealth Security

**DOI:** 10.12669/pjms.304.5210

**Published:** 2014

**Authors:** Mohammad Bajwa

**Affiliations:** Mohammad Bajwa, PhD, Program Director, Healthcare Systems Management, Metropolitan College of New York, 431 Canal Street, NY 10013, USA.

**Keywords:** Electronic Health Record (EHR), Electronic Medical Record (EMR), Radio Frequency (RF), Smart phones, Tablet Personal Computers, Laptop Computers, Personal Digital Assistants (PDAs), Global Positioning System (GPS)

## Abstract

With the implementation of Electronic Health Record (EHR), the patients’ health information will reside on the network of computers that can be accessed through a variety of techniques and technologies. One of the widely used present-day communication technology is the mobile/cell phone that has great potential in the healthcare delivery and management as the healthcare providers can remotely access electronically stored health information of patients from anywhere anytime. One of the greatest advantages of the mobile health technology is convenience of time-independent communication from geographically distant places with the healthcare centers and patients that enhances healthcare quality at reduced cost. However, its equally great disadvantage lies in sending and receiving the health information wirelessly through atmosphere which can be easily intercepted, eavesdropped, interjected, modified or even destroyed.

## INTRODUCTION

Mobile technology has changed the way we live, work and communicate. It is a type of wireless technology that implies sending and receiving messages by electromagnetic (EM) signals, principally radio waves, through the atmosphere instead of the wired media using copper or fiber optic cables. Having emerged only in 1980s, it is the latest and the least understood communication technology.^1^^,^^2^

Use of mobile health technology, commonly referred to as mHealth, in the delivery and management of healthcare, is a very recent development having only been introduced in the 21^st^ century and is still in infancy. But it is gaining impetus due to the world wide implementation of electronic health records (EHRs) whereby the patients’ health information will reside on computers and travel on computer networks. Normally the information stored in computers is accessed through wires and hence limited to places where such infrastructure exists. But with the advent of wireless technology, computers can be accessed by anybody, anytime and from anywhere so long as wireless connectivity is available.^3^

Despite this advantage, security of the health information stored on and accessed through mobile devices is a huge concern as wireless communication is prone to be hacked and mobile devices being small and valuable are liable to be misplaced, lost or stolen.

The intent of this brief treatise is to review the development of mobile technology, its potential uses in healthcare, barriers to adoption, security concerns, and ways and means to ensure security of the electronic protected health information (ePHI) accessed through mobile devices. 

**MOBILE HEALTH DEVELOPMENT**

Mobile Health or mHealth is the use of mobile devices (mDevices) in the practice of medicine. These include mobile/cell phones, iPads, tablet, personal, and laptop computers, personal digital assistants (PDAs), and similar other devices that use wireless technology to access the health information networks. Such devices often use radio waves for communication either through central access points (hot spots) or satellites and of these the mobile phones have emerged as the greatest mobile technology.^4^

The use of mobile phones started in early 1970s and their use was restricted to voice communication only. They were bulky, expensive, had low life batteries lasting a few hours, and their connectivity was sparse depending upon the availability of company’s network infrastructure, resulting in frequent call drops and service unavailability. Since then, the mobile phones have shrunk both in size and price with enormous expansion in geographical reach and functionality from audio to text, image, e-mail and video communication with built-in cameras and global positioning systems (GPS). The ordinary (dumb) phones thus have transformed to smart phones and their increased functionality has greatly enhanced their potential use in healthcare.^5^ A wide variety of branded devices (iPhone, Blackberry, Android, Windows phone, etc.) with these features is now available. The latest being the tablet personal computer (Tablet PC) that is larger in size and can perform less resource intensive computer functions also.

**USE OF MOBILE TECHNOLOGY IN HEALTHCARE**

Mobile devices now come in all sizes and shapes and range from those that can fit hands, pockets, and purses.^6^ However, the smart phones and tablets account for the most and have found their way from personal to enterprise-wide use. They were originally designed for the consumer market, but vendors overtime have added enterprise-friendly applications to them. Because of their roots in the consumer market, they have several security loopholes for enterprise and healthcare use.^7^

***Smart Phones: ***A smart phone with internet access and other built-in features like text messaging, e-mailing, Web browsing, camera, GPS, and audio and video, etc., is the latest development of the mobile/cell phone family. Smart phones are more of handheld computers as they can install and run several applications. Many of these now have touchscreen features, large screens, physical keyboards, large memory and powerful processors. Another big attraction of smart phones is the availability of large number of third party applications which consumers can download for a nominal fee or free. Since these devices are now exposed to large networks, like computers, they are susceptible to all types of cyber-attacks and hence their security is emerging as a big issue.

***Tablets: ***A tablet PC is a wireless, portable personal computer with a touch display screen that also acts as input device. The tablet is typically smaller than a notebook computer but larger than a smart phone. Since they are small and valuable, and are apt to be lost and stolen, their physical security poses significant problems. Their wireless communication without the security features, like encryption, still remains a significant concern. Despite these deficiencies, they are becoming increasingly popular among the healthcare providers.

**mHEALTH NETWORKS**

mHealth has recently and largely emerged in the developing countries due to cheap availability of the cell phone technology. The major uses include collecting clinical and health data; delivery of healthcare information to practitioners, public, researchers, and patients; real-time monitoring of patients’ vital signs; and direct provision of care via telemedicine.^8^

The mobile/cell phones work on Wireless Wide Area Network (WWAN) that uses cell towers to connect devices and transfer communications within the area of cellular network.^9^ Having progressed from 1G to 5 G (first to fifth generation), these devices can now perform multifarious functions using broadband capabilities to transfer large chunks of multiform healthcare data.^10^ Another wireless network technology emerged recently is the Wireless Private Area Network (WPAN) for communication within personal space using Infrared (IR) spectrum (IR scanners) and low energy radio waves (Bluetooth technology). These technologies afford communication with devices in one’s personal reach.

**BENEFITS OF mHEALTH TECHNOLOGY**

The mDevices offer remarkably attractive low cost, real-time access to dynamic information on diseases, disorders, behavior, social interactions, environmental toxins, metabolites, and a host of other physical and physiological variables.^11^ Collins^10^ narrated his successful experience from a voluntary participative study where cell phones with sensors were used to monitor participants’ health with respect to chronic diseases like diabetes, hypertension, and other medical conditions such as heart rate and rhythm, and even recording and transmitting EKG. These observations are supported by McKenna^12^ and Blondel^13^, who opined that cell phones could become weapons against diseases. Talbot^14^ reported that cell phones were great sources of health data. Similar ideas have been forwarded by the Center for Technology and Aging^15^ in a draft paper emphasizing that mobile devices would be extensively used for monitoring the health of senior citizens by 2014. Not only this, but smart watches (watches that can perform computerized functions and run applications like cellphones) will be used to display selected data from the cell phones so that medically critical information can be seen at a glance ^16^ without taking cell phones out of pocket.

**BARRIERS TO mHEALTH**

Major barriers to the adoption of mHealth are resistance to innovation, lack of infrastructure, and cost of technology acquisition and ownership.^17^ One of the major concerns about the use of mHealth is the security of the health information being accessed through and residing on mobile devices. mHealth provides mobility and remote connectivity, but it also brings in significantly more security threats than the traditional wired networks. Other policy barriers to mHealth are cost of implementation and infrastructure maintenance, liability, and security issues. HIMSS^11^ has postulated additional obstacles as lack of business model, security, standards, and regulatory compliance guidelines for mHealth.

**mHEALTH SECURITY THREATS**

The advent of wireless technology and its widespread adoption in healthcare is scary because mHealth uses wireless atmospheric media to transmit information as radio signals which are vulnerable to eavesdropping, modification, theft, distortion and even loss. Disturbingly, majority of the mDevices, especially the smart phones, possess only rudimentary security settings making them vulnerable to hackers seeking access to patients’ ePHI (electronic personal health information), EMR (electronic medical record)/EHR (electronic health record), financial, and other sensitive information stored on the healthcare facility’s networks. The mDevices being miniature in size are also liable to be misplaced and lost, endangering disclosure of any confidential information stored on them or accessed through them. Many free tools on the web are now available to hackers that can break the weak security measures, like Wired Equivalent Privacy (WEP) security protocol, commonly employed to secure mDevices, like cell phones. The security even becomes more threatening when clinicians start using their own mDevices, called BYOD (bring your own devices) accessing patient information from the healthcare providers’ network. No doubt with such a facility, clinicians are approachable anywhere and anytime and can also make and convey faster medical decisions for better patient outcomes, this seductive technology is not without its risks as it can open up new ways for the health information to be compromised. Choi^18^ opined that the embedded computers, like cell phones, are very vulnerable to attacks because they are highly networked with other wireless devices and because they have virtually no defenses against protecting their firmware and programs hardwired into the chip. The most common mobile device security threats include:^7^

***Loss, theft and replacement: ***The mobile devices due to their small size can be lost or slide out of pocket, stolen or given for replacement or upgrades.

***Off-site data storage: ***Ever expanding storage capabilities of mobile devices leading to storage of corporate sensitive data on mDevices.

***Network access outside your control: ***Mobile devices can connect to any network outside the company’s control. This proliferation of wireless interfaces exposes ever-increasing attack surface that can be used to compromise mobile devices by the attack vectors.

**mHEALTH SECURITY MITIGATION**

As mobile technology enters the healthcare arena, information technology administrators are faced with several unique challenges like: With which mDevices users can access health information? How would they authenticate to the server? Which data should be allowed to be stored on the mDevices? How should misplaced or lost devices be recovered? How should data on the lost or misplaced devices be erased? Which security protocol should be employed for which medical application? And how should data breaches be handled?

As a result, several different strategies have been proposed to alleviate vulnerabilities of mHealth devices and avoid risks of security incidences. These include performing security risk assessments, setting security goals, and developing and executing security strategies and tactics.^19^ Few mHealth security measures proposed by McNickle (2012)^20^ are:

1. Use geolocation software or services to track, locate, or wipe the device of data.
2. Brick (clean all data and software) the device if it is lost or stolen.
3. Encrypt all devices that store health or health-related information.
4. Configure to automatic encryption and decryption only on authentication.
5. Use strong safeguards to permit access to ePHI through mDevice.
6. Educate employees on the importance of safeguarding their mobile devices.
7. Purchase only those networkable devices that have well-documented and fine-grained security features and the medical IT network engineers can configure safely.
8. Include in the purchasing document vendor support for ongoing firmware, patch and antivirus updates suitable for risk mitigation strategy.
9. Operate well-maintained external facing firewalls; implement network monitoring and intrusion detection technologies, and segment network containing medical devices.
10. Configure Access Control Lists (ACLs) on the network segments for only the positively authorized accounts to access.
11. Establish strict policies for the connection of any networked devices to health information network.
12. Establish policies to maintain, review and audit network configuration as routine activities when the medical network is changed.
13. Use principle of least privilege to decide which accounts need access to specific medical device network segments rather than providing access to entire network.
14. Implement safe, effective, legal patch and software upgrade policies for medical IT networks which contain regulated medical devices.
15. Secure communication channels, particularly wireless by the use of encryption and authentication at both ends of communication channels.
16. Enforce password policies, and
17. Use VPN (Virtual Private Network) to access the corporate network over the Internet.

## CONCLUSION

In conclusion, the explosive use of mDevices in the healthcare arena has several benefits including accessing the health information from anywhere at any time, delivering and monitoring healthcare, and educating and communicating with the patients. However, mobile technology has also brought serious security concerns as the wireless information travelling through the atmosphere is prone to be intercepted, interjected, modified, or even destroyed. Since the mDevice popularity for healthcare cannot be halted, adoption of best practices, such as strong authentication and encryption standards and protocols for securing health information stored on and/or accessed by these devices, is the best solution. Education and training of users and making them security cautious, should be used as first line of defense in securing the mHealth information. Creation of security culture among the users of mDevices would particularly yield rich dividends.
